# Molecular Correlates of Social Dominance: A Novel Role for Ependymin in Aggression

**DOI:** 10.1371/journal.pone.0018181

**Published:** 2011-04-05

**Authors:** Lynne U. Sneddon, Rupert Schmidt, Yongxiang Fang, Andrew R. Cossins

**Affiliations:** 1 Institute of Integrative Biology and Centre for Genomic Research, University of Liverpool, Liverpool, United Kingdom; 2 Zentrale Biotechnische Betriebseinheit, University of Giessen, Giessen, Germany; Cajal Institute, Consejo Superior de Investigaciones Científicas, Spain

## Abstract

Theoretical and empirical studies have sought to explain the formation and maintenance of social relationships within groups. The resulting dominance hierarchies have significant fitness and survival consequences dependent upon social status. We hypothesised that each position or rank within a group has a distinctive brain gene expression profile that correlates with behavioural phenotype. Furthermore, transitions in rank position should determine which genes shift in expression concurrent with the new dominance status. We used a custom cDNA microarray to profile brain transcript expression in a model species, the rainbow trout, which forms tractable linear hierarchies. Dominant, subdominant and submissive individuals had distinctive transcript profiles with 110 gene probes identified using conservative statistical analyses. By removing the dominant, we characterised the changes in transcript expression in sub-dominant individuals that became dominant demonstrating that the molecular transition occurred within 48 hours. A strong, novel candidate gene, ependymin, which was highly expressed in both the transcript and protein in subdominants relative to dominants, was tested further. Using antibody injection to inactivate ependymin in pairs of dominant and subdominant zebrafish, the subdominant fish exhibited a substantial increase in aggression in parallel with an enhanced competitive ability. This is the first study to characterise the molecular signatures of dominance status within groups and the first to implicate ependymin in control of aggressive behaviour. It also provides evidence for indirect genetic effect models in which genotype/phenotype of an individual is influenced by conspecific interactions within a group. The variation in the molecular profile of each individual within a group may offer a new explanation of intraspecific variation in gene expression within undefined groups of animals and provides new candidates for empirical study.

## Introduction

Individual fitness is driven by the acquisition of key resources necessary for survival and reproduction. Social status often plays a crucial role in gaining these resources, with dominant animals monopolising or having priority access, and rank within a social group having a profound effect upon reproductive success, survival and ultimately fitness [1]. Dominance status correlates with a suite of behavioural and physiological parameters. Thus, dominant individuals are more willing to perform aggressive attacks [2], [3], [4], [5]. They also have lower stress hormone levels, differing brain serotonergic activity, more efficient metabolic and growth rates than those measured in subdominant and subordinate animals [6]. Usually these parameters are determined sometime after a dominance hierarchy has been established and, therefore, it has been difficult to separate cause and consequence. Understanding the molecular basis of the aggressive behaviour that underlies social status helps define the extent to which individuals vary physiologically within groups, since the dominance status of individuals is generally not accounted for in molecular and physiological studies and likely contributes to the observed variance. Furthermore, a mechanistic approach may identify indirect genetic effects such as phenotypic traits of conspecifics that contribute to individual fitness to explain the evolution of complex social groups [7].

To date few attempts have been made to correlate dominance status with gene expression profiles in groups of animals. Contemporary post-genomic screening technologies now offers an efficient means of identifying large numbers of genes whose expression correlates with complicated behaviours. These have yielded important insights into life history patterns in Atlantic salmon, *Salmo salar* [8] identifying genes that differ between alternative mating strategies, as well as those genes correlated with social plasticity and gender in a cichlid, *Astatotilapia burtoni* [9]. Other behaviours such as division of labour [10] and response to alarm pheromone in honeybees [11], seasonal changes in territoriality in songbirds [12], propensity to aggressively peck in chickens [13], geotaxis in *Drosophila* [14] and learning and memory in mice [15] have been linked to specific genes using transcript profiling. However, few of these studies have tested the candidate genes identified from these microarray screens to support a causal relationship between gene expression and behavioural performance [16].

Here we have compared the gene expression profiles of dominant, sub-dominant and submissive rainbow trout, *Oncorhynchus mykiss*, using a custom-built cDNA microarray. Trout form robust, tractable linear dominance hierarchies, through easily quantifiable aggressive interactions [17]. Furthermore, much is known about the distinctive behavioural and physiological differences that relate to social status [6], [18]. We have previously established that gene expression profiles were correlated with dominance status, but individual genes were not identified or studied further [17]. We also test whether those genes displaying correlated expression properties also showed changes in expression during the experimentally-induced transition of individuals between status levels. Together these two approaches have identified a new candidate gene, ependymin, and its encoded extracellular protein, which have not previously been linked to aggression. Ependymins are secreted by meningeal cells and are the predominant glycoproteins in the cerebrospinal fluid (CSF) in teleost fish. Studies have shown injection of antisera intracerebroventricularly results in decreased brain ependymin protein levels and deficits in learning and memory recall in zebrafish [19], [20]. Therefore, ependymin is secreted into the CSF and reuptake into the brain can be prevented by inactivating ependymin using the antisera injection technique. Using antisera to bind and inactivate the expressed ependymin protein in the zebrafish, *Danio rerio*, we have tested whether this protein is linked to aggressive interactions.

## Results

### Transcript profiling of brain gene expression in stable and manipulated dominance hierarchies

We fabricated a cDNA microarray composed of 11,047 EST-sequenced cDNA clones generated from normalised cDNA libraries prepared from brain, liver and skeletal muscle. Of these, 1762 ESTs were annotated by homology alignment, representing 494 unique genes identified by BLASTx searching of which 454 possessed Gene Ontology annotation. This microarray was used in two experiments. First, we determined the transcript expression profiles for brain RNA from the dominant, sub-dominant and submissive specimens from each of 6 stable replicate hierarchies, using a reference-based experimental design based on single pool of reference RNA from all samples. Second, we explored the changes of gene expression in manipulated hierarchies. For this we set up 6 replicate hierarchies, in which replacement of the dominant fish with an approximately 30% smaller specimen led to the previously sub-dominant fish adopting the dominant's position. Brain RNA was isolated from each of these newly dominant specimens at 3 time points (2 h, 48 h and 1 week) after removal of the original dominant fish, giving 18 RNA preparations. Again each preparation was analyzed against the common reference RNA.

**Figure 1:**
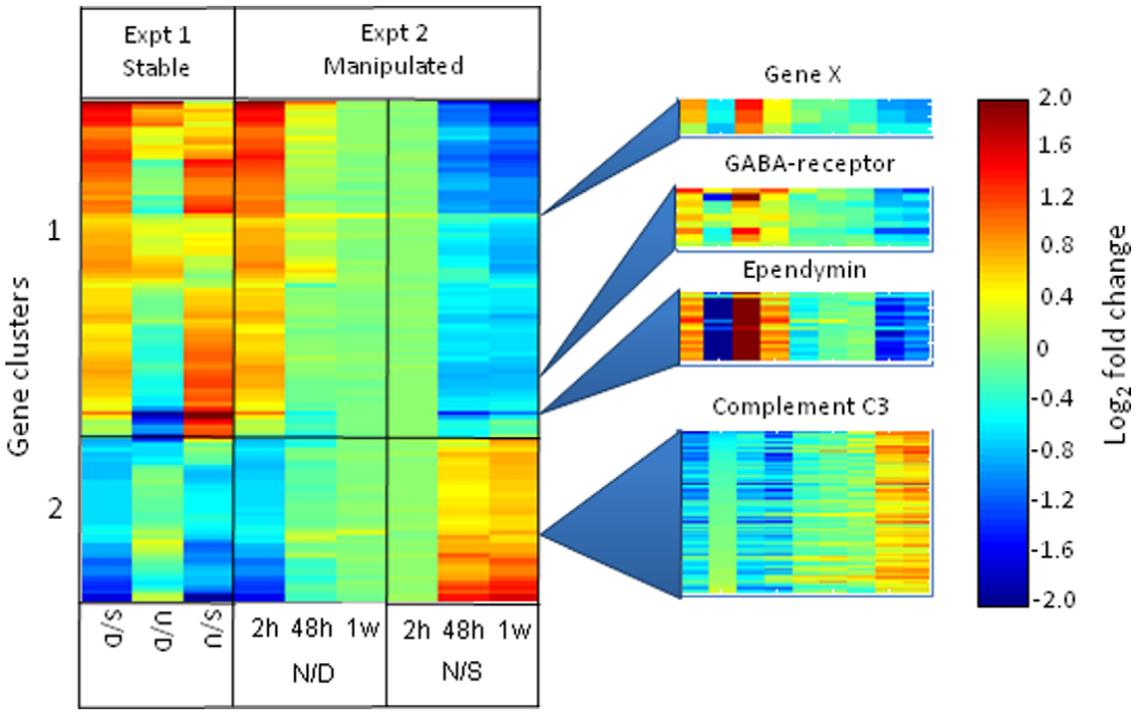
Transcript expression in stable and manipulated rainbow trout dominance hierarchies. A colour-coded expression profile for genes that were differentially expressed in the brain between dominant (D), subdominant (S) and subordinate (U) rainbow trout taken from replicated stable hierarchies. Values represent log_2_ fold-change values between contrasted treatments (D  =  dominant, S  =  subdominant, U  =  subordinate) with a colour coding indicated in the panel to the right. 110 differentially expressed genes were hierarchically clustered into 2 groups according to the outcome of Experiment 1. Experiment 2 displays the effects over a 7-day period of hierarchy manipulation by replacement of the original dominant fish with a smaller fish. The left-hand group (N/D) shows the changing profile of original subdominant fish (N) relative to the dominant previously determined in the stable hierarchies in Experiment 1 (D), whilst the right-hand group (N/S) compares the original subdominant fish with the stable dominant. Each row in the main panel represents a single differentially expressed gene probe selected from those included on the microarray for a given BLASTx identity. The four smaller panels to the right display the expression profiles for all differentially expressed gene probes for ependymin (19 clones), GABA-RAP (9 clones), Gene X (3 clones) and for one of 5 expression clusters for complement-C3 (45 clones). This Figure shows how social status is linked to distinctive transcript profiles. Removal of the dominant in Experiment 2 resulted in the previously subdominant assuming dominance which was reflected in the brain transcript expression that changes within 48 hours to a profile characteristic of a dominant.

For the purposes of defining the list of differentially expressed genes, we adopted the following conservative criteria; a false discovery rate (FDR) of 10% (q = 0.1) and a fold-change difference value of greater than 1.5. This revealed 110 unique brain genes (Table S1) responding in either of the two experiments for at least one of the statistical comparisons. The combined responses were K-means clustered (see the ‘heatmap’ in Figure 1) across all data generating 2 major gene clusters, cluster 1 which for Experiment 1 was up-regulated in the sub-dominant group (S) relative to both the dominant (D) and the subordinate (U) specimens, and cluster 2 which showed the reverse. The contrast of U with D displayed much smaller fold-change differences for most genes, the main exceptions being ependymin and phosphoglycerate kinase.

**Figure 2:**
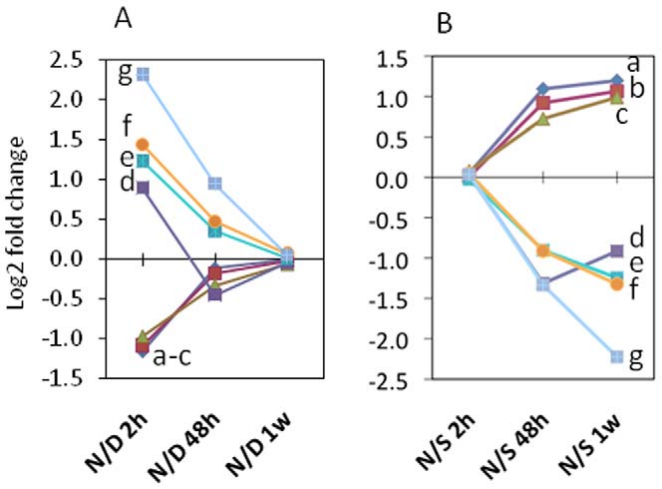
Temporal expression of selected genes during the transition from subdominance to dominance in rainbow trout. Changing transcript expression of 7 selected genes following manipulation of social hierarchy by adoption of dominant (D) status by the previously subdominant (N) fish. Panel A displays the fold-change in expression of the newly dominant fish relative to the original dominant fish (N/D) at three times (2 h, 48 h, 1 week) after removal of the original dominant. Panel B displays the corresponding contrast between N and the subordinate fish. Gene identities: a – Ca++ transporting ATPase; b – ubiquitin A52 residue ribosomal fusion product; c – ribosomal protein L32; d – ependymin; e – claudin-3; f – mitochondrial H+ transporting ATP synthase F1; g – phosphoglycerate kinase 1. Panel A demonstrates that the transcript expression in the brains of subdominants assuming dominance status is changing to become similar to a stable dominant's profile. In contrast, Panel B illustrates that the new dominant is becoming more dissimilar to the subdominant profile. These changes occur within the 48 hour time period.

In the second, manipulation, experiment the previously subdominant specimens that became dominant (N) from 2 h, 48 h and 1 week, were separately contrasted with the dominant profile from Experiment 1 as indicated in the N/D contrasts in Figure 1 (see Figure 2A for representative genes and Figure S1A for the full set). They were also contrasted with the subdominant (S) profile from the stable hierarchies experiment (N/S contrasts in Figures 1, 2B and S1B). At 2 h after removal of the dominant the N/D profile broadly matched that of the S/D profile in Experiment 1. At 48 h and 1 week the difference reduced in magnitude, as the newly dominant specimens progressively adopted a profile characteristic of the stable dominant fish.

By contrast, for the N/S comparison the differences were non-existent at 2 h, but increased by 48 h until they broadly matched that expected for the D/S contrast (reciprocal of S/D contrast in Figures 1 and 2B). Together these results demonstrate that the differentially expressed genes identified from the stable hierarchies displayed the expected transitions when sub-dominant fish assumed a dominant status, and that the main changes in transcript expression occurred between the 2 h and 48 h time points after manipulation.

The 110 unique DE genes that possess functional annotation are listed in Table S1 within broad functional and cell compartment categories. For protein turnover, we detected a general up-regulation in the subdominant of 8 out of 9 ribosomal proteins (the exception being L32), a translation elongation factor, two 26S proteosome subunits, and a dipeptidase and serpin peptidase inhibitors, compared to both dominant and subordinate groups. Cathepsin Z precursor, a lysosomal protease, and a ubiquitin ribosomal fusion protein were down-regulated. Of the stress proteins, hsp90a and b were both up-regulated in the subordinate compared to the other ranks, whilst hsp40 (DnaJ) was down-regulated. We detected changes in expression of two important active transport systems: thus two Ca^2+^-transporting ATPase isoforms and a cadmium translocating P-type ATPase were both down-regulated in the subordinate compared to the other groups, whilst the Na^+^/K^+^ ATPase a and b subunits showed the reverse. In energy pathways, we found increased expression in the subdominant of two H+ transporting F1 ATP synthase subunits, creatine kinase, succinate CoA-ligase, and glycolytic genes including fructose 1,6 bisphosphatase, phosphoglycerate kinase and mutase. By contrast, a cytochrome c oxidase subunit and a succinate CoA ligase subunit were down-regulated. A number of cytoskeletal proteins were up-regulated in subdominant fish including a two myosin heavy chain isoforms, tropomyosin and tubulin proteins, and adhesion proteins such as ERGIC, cadherin and catenin and claudin. Yet collagen and a myosin light chain subunit were down-regulated. For genes involved with cellular-level regulation we found that several calmodulin genes were up-regulated in subdominant fish, whilst three regulatory genes (retinoic acid binding protein, proteinase-activated receptor 2 and a signal sequence receptor, were down-regulated. For nuclear regulation, we identified two up-regulated high mobility group genes as well as several up-regulated transcriptional regulators including polyhomeotic-like and PalB-like genes.

**Figure 3:**
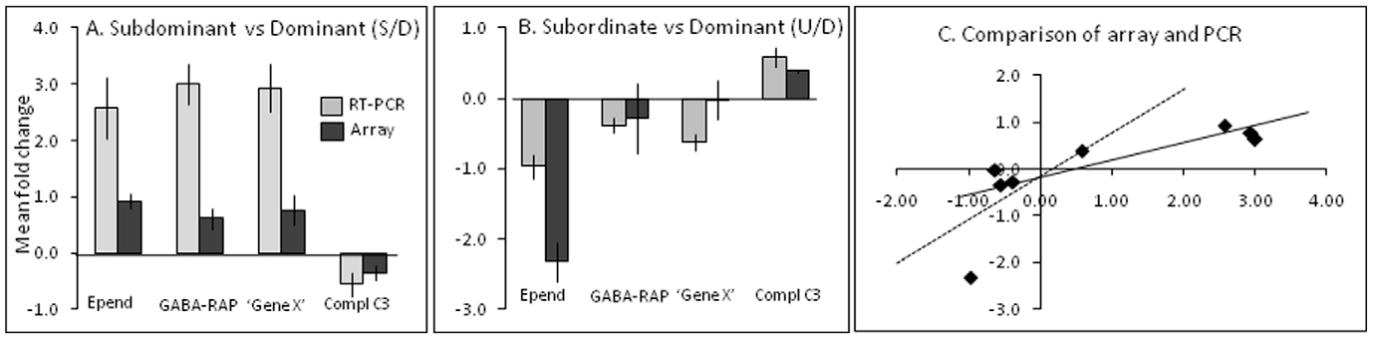
RT-PCR validation of the microarray results. Confirmation of the array expression profile of four selected genes that were significantly different between the subdominant (A) and the subordinate (B) when compared with the dominant (n = 6). Mean fold change values (±SE) from the microarray and RT-PCR data are compared for ependymin (epend), GABA receptor (GABA RAP), Gene X, and complement C3-1 (Compl. C3). Gene X is an unidentified clone (Genbank CA964433) with an exact match with cDNA clone TC18973 from *Ictalurus*, and homology with ESTs from rainbow trout (CA377677.1), and a zebrafish ORF (zgc:73352) which has been linked to clusterin. Panel C relates fold changes values of the two measures with the array value plotted on the vertical axis and the RT-PCR value on the horizontal axis. The dashed line represents equality. The RT-PCR measurements confirm the results from the microarray experiment except that the array results underestimate the fold differences.

Four candidate genes from the array analysis were selected for verification by RT-PCR, namely ependymin, GABA-receptor-associated protein (GABA-RAP), complement-C3 and an unidentified gene, termed Gene X, with homology with brain genes found in other fish species linked to MAP kinase activity. Figure 3A and B contrasts the subdominant with dominant, and subdominant with dominant, respectively. The RT-PCR data broadly confirmed the array-based differences between hierarchies. Thus, ependymin, Gene X and GABA-RAP were all up-regulated in subdominant and down-regulated in subordinate, all relative to the dominant. Also complement C3-1 was down-regulated in the subdominant and up-regulated in the subordinate, both relative to the dominant. However, the array data displayed much lower fold-change values compared to the PCR, as displayed in Figure 2C.

Ependymin displayed the largest differences between members of the stable hierarchy; thus, the subdominant was 6.5-fold greater than in the subordinate group and 1.6-fold greater than the dominant group. It also showed the largest changes in expression during the manipulation experiment. Ependymin is a protein secreted into the cerebrospinal fluid by the meninges and uptake by the brain occurs from the extracellular fluid [19], [20]. Using a western immunoblot and an anti-ependymin antibody we have compared the expression of ependymin protein in the brains of dominant, subdominant and subordinate trout. Densitometric values for each protein extract were normalised to β-tubulin as reference gene (Figure S2). The mean expression levels for ependymin were 0.88±0.13 for the dominant, 3.89±0.06 for the subdominant and 0.46±0.09 for the subordinate (F_2,6_ = 5.94, P<0.001).

### The role of ependymin in aggressive behaviour

Previous work has established in zebrafish that expression of extracellular ependymin can be inactivated by direct intracerebroventricular injection of the antiserum, with maximal down-regulation at 6-8 hours, after which recovery occurs [19], [20]. We used this approach to explore the relationship of manipulated ependymin expression with behaviours associated with establishing the dominance/sub-dominance relationships. We compared the rates of aggressive chases and the proportion of food obtained by dominant fish and subordinate fish both before and after sham treatment (anaesthesia but no injection), and after intra-cerebroventricular injection of either phosphate buffer saline (PBS) or PBS containing the antiserum.

**Figure 4:**
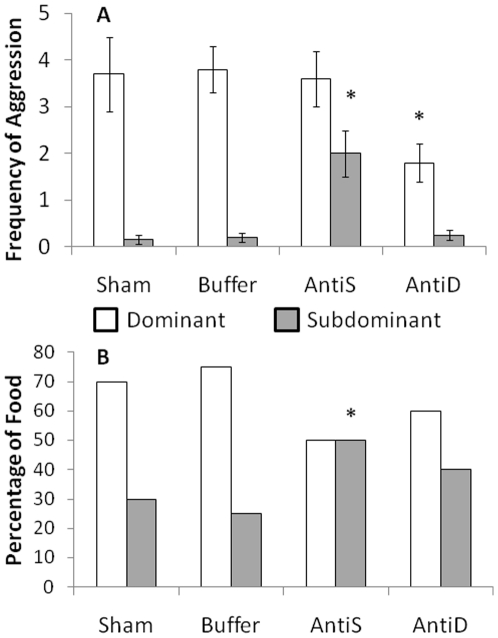
Impact of inactivation of ependymin in dominant and subdominant zebrafish. (A) The mean frequency (±SE) of aggressive attacks performed by the dominant and subdominant zebrafish in the sham control (Sham), subdominant injected with buffer (Buffer), subdominant injected with antisera (AntiS) and the dominant injected with antisera (AntiD) after treatment. The treatment was applied 6 hours before observation which relates to maximal inactivation of ependymin (19,20) when antiserum was applied. The subdominant injected with antisera (AntiS) showed a significant increase in aggressive attacks compared with its normal behaviour prior to treatment and when compared with the control groups (*P<0.001). The dominant injected with antisera (AntiB) exhibited a significant decrease in aggression (*P = 0.023). (B) The median percentage of food acquired by the dominant and subdominant zebrafish in the Sham Control (Sham), Subdominant injected with buffer (Buffer), subdominant injected with anti-sera (AntiS) and the dominant injected with antisera (AntiD) after treatment. Again the treatment was applied 6 hours before observation 3 where the subdominant injected with antisera (AntiS) obtained significantly more food compared with the control groups (*P = 0.021). Therefore, ependymin inactivation led to a significant increase in aggression and competitive ability in subdominant zebrafish. In contrast, ependymin inactivation led to a decrease in aggression in dominant zebrafish.

Before injection the dominant was much more aggressive (ANOVA F_1,21_ = 293.7, P<0.001) and obtained a greater proportion of food (Kruskal Wallis H = 307.6, df = 1; P<0.001) compared with the subdominant fish. After treatment the aggressive behaviour and the percentage of food eaten were unaffected by injection of buffer or by the sham-injected control (Figure 4). However, administration of ependymin antisera into the subdominant fish increased their aggressive behaviour (T = 25.37; P<0.001; n = 8) and the amount of food consumed (W = 36.0, P = 0.014) immediately after treatment. In contrast, the antisera treated dominants exhibited reduced aggression (T = 3.25; P = 0.023; n = 6) but food acquisition was unaffected (W = 0, P>0.05).

When comparing between the subdominants in each group, aggression was highest in the antisera treatment group (F_3,25_ = 15.65, P<0.001; Figure 4A) coupled with increased food intake in subdominant (H = 9.56, P = 0.021; Figure 4B). Aggression was much lower after antisera treatment in the dominants when compared with dominants from the other groups (F_3,25_ = 3.44, P = 0.032; Figure 4A), however, food intake was not affected (H = 2.65, P>0.05; Figure 4B) The effect of antisera administration on aggression and food intake was transient since behaviour returned to the pre-administration state at subsequent observations (24 h) with the dominant again being more aggressive and obtaining the greatest proportion of food.

## Discussion

To our knowledge this is the first study to assess global gene expression in relation to the complicated behaviours involved in the establishment and maintenance of dominance hierarchies within groups of individuals. Most studies have examined pair-wise states such as reproductively active males versus those of lower status [9]; sneaker males versus alpha males [8]; responses to intruders in and out of breeding season [12] or between high and low aggressors [13]. Although these have yielded important information, these animals do not generally live in discrete pairs and the evolution of social groups should be considered by studies considering how phenotype/genotypes of individuals interact with one another at the genetic level [7]. Here we have provided clear evidence for the “indirect genetic effects” (IGE) model which states that traits are not only influenced by an individual's own genetic make-up but also by the genotype of the conspecifics that the individual interacts with [7], [21]. A common but largely unappreciated problem in functional genomic studies of out-bred animals is accounting for the variation observed between individual specimens within a population or treatment group [9]. Some of this variation is clearly of genetic origin, but a component might also be due to differences in social status, either due to the activation of genes that directly mediate social dominance, or genes responding to the contingent differences in stress hormone activation, the rates of food acquisition and of protein turnover, etc. Identifying the gene regulatory signatures of social status might, therefore, reduce uncertainty in genomic-level analysis and interpretation. They also might point to the mechanisms accounting for the observed differences in aggression and physiology, at least in salmonid fish [22], [23] and in other species that form linear hierarchies [1], [24].

We now show that the brain transcriptomes for trout of different social status were distinctive, with the largest differences evident when contrasting sub-dominant with either dominant or subordinate trout. Moreover, experimentally-induced transitions of individuals in status from sub-dominant to dominant led to changes in gene expression profile converting from a typically sub-dominant pattern to a dominant pattern demonstrating that dominance status is a product of the interaction of conspecifics and as such fits within the IGE model [7]. These changes were substantial and almost complete within 48 h, and any differences between the newly dominant fish and the previous dominant from the stable hierarchy had entirely disappeared at 7 days after removal of the dominant. This outcome applied to both up- and down-regulated clusters of genes. Salmonid fish are known to establish dominance relationships in just a few hours [24], [25], which suggests that the changing transcript profiles measured at 48 h and 1 week are linked in both direction and time to social status. Those molecular changes which occurred much more rapidly between 2 and 48 h are crucial for identifying novel genes underlying the establishment of dominance and associated changes in behaviour and physiology. These rapid changes in brain transcript profile may mean that molecular changes linked to social status are dynamic and possibly reversible. Unfortunately the terminal sampling procedure precludes us from testing this directly but it is likely that dominants who fall in status will also exhibit a profound change in their transcript profile correlated with their lower status.

We used conservative statistical criteria to identify 110 unique genes that displayed differences in expression between hierarchical groups. This list represents approximately 33% of the unique, annotated genes represented on what was a comparatively small cDNA array. In the subdominant, genes with functions in protein turnover, metabolism, cell structure and transport and stress were up-regulated and may reflect changes in energy expenditure when engaging in fights since this rank is known to lose weight over the experimental period [4], [17], [26]. These animals may be metabolically compromised resulting in the breakdown of proteins and increased metabolism due to reduced energy availability. Moreover, development of these individuals may be impaired due to low energy intake or poorer feed conversion efficiency resulting from the stress of their social position [27]. Complement C3 has an immune function and is down-regulated in response to stress [28] which possibly explains why it is down-regulated in the most stressed member of the hierarchy, the subdominant. GABA-RAP has been linked to aggression in a variety of species including humans, since GABA appears to be of particular significance in the neurochemical control of aggressive behaviour [29]. Whilst gene ontology enrichment analysis failed to identify any enrichment of GO categories we have identified groups of responding genes involved in protein turnover, intermediary and energy metabolism, and regulation at the level of nuclei, cells and systems. However, of all the differentially expressed genes, ependymin was a clear candidate and stood out as having the largest difference in expression levels between hierarchical levels, with ∼2-fold higher levels in sub-dominant than dominant, a 6.6-fold difference between sub-dominant and subordinate, and a 5.5-fold larger expression in the subordinate compared to the dominant.

Ependymin is a brain neurotrophic factor originally identified in the cerebrospinal fluid of teleost fish, but now known to be a member of a larger gene family distributed widely across vertebrates, invertebrate deuterostomes and protostomes [30]. In fish brain it functions in a variety of cellular events related to long-term memory, neuronal regeneration and adhesion [19], [20], [31]. This encoded protein correlates with behavioural performance at the neuronal level especially in the formation of long-term memory [19] but also to environmental stress [32],[33]. Whilst ependymin has not previously been linked to aggression or to status in a social hierarchy, it is more highly expressed in the brains of sneaker male trout that adopt an alternative mating strategy since they cannot out-compete large, dominant males [8]. Thus, up-regulation of this gene was linked to lower competitive ability, which is consistent with the present results with lower expression in dominant relative to sub-dominant fish.

Using an anti-ependymin antibody, we have shown in trout that the encoded protein also displays changes in expression in concert with whole brain transcript abundance. Thus, we found a 4.5-fold increase in protein expression in the sub-dominant group compared to the dominant, and a two-fold greater amount in the dominant compared to the subordinate. Direct intracerebroventricular injection of the anti-ependymin antibody has previously been used to manipulate expressed ependymin levels in the zebrafish, *D. rerio*, and this resulted in impaired learning and memory recall [19], [20]. This confirms that the injection technique was effective in this species and that some behavioural properties were influenced by inactivation of the available protein. We demonstrated that zebrafish when housed in pairs also display aggressive interactions between individuals and that the dominant individual obtains the greatest proportion of food. Sham-injected and control zebrafish were unaffected by treatment as indicated by measurement of the rates of food acquisition or of aggressive attacks. By contrast, injection of the subdominant with anti-ependymin antibody resulted in a significant increase in aggressive behaviour and an increase in the proportion of food obtained. Ependymin inactivation in the dominant led to a decrease in aggression, however, food acquisition was unaffected. These effects were transient in the dominant and sub-dominant fish, with aggressive behaviour returning to normal 24 h after treatment, presumably due to continued transcription and restoration of ependymin protein [19], [20].

The direct modification of a trait by manipulation of a single gene product constitutes *prime facie* evidence that the gene and its encoded protein are contributory or causal factors affecting the behavioural phenotype and the outcome in terms of dominance status. Thus, the observed effect of anti-ependymin treatment on social behaviour in the zebrafish model thus points to a causal link between ependymin expression and aggressive behaviour, and to the generation of a social hierarchy. Moreover, this effect is entirely consistent with the observed differences in expression of both ependymin transcript and protein in subdominant and dominant trout and also with the effects of dominance manipulation on ependymin expression. But whilst this suggests a novel function for ependymin the relationship between ependymin expression and social status was not simple; ependymin protein level in trout decreased in the order subdominant>dominant>subordinate. Thus ependymin has a U-shaped relationship with hierarchical rank, such that both low and high levels of expression relate to low aggression, whereas the rank 1, dominant trout have intermediate levels of expression. This is confirmed in the zebrafish experiments; dominant fish became less aggressive when ependymin was inactivated and their protein levels would have been reduced to those seen in subordinates who behave more submissively. However, when ependymin was inactivated in subdominant zebrafish, this may have reduced their protein levels to those seen in dominants resulting in increased aggression.

Ependymin has previously shown to be involved in memory consolidation [34] and the enhanced expression of ependymin in subdominant fish may be linked to specific behavioural properties of this hierarchical level. Given its position in the hierarchy, the subdominant fish needs to be most attentive to avoid provoking the dominant, but it has to fight with the dominant to gain access to food or space. Thus, the temporary increase in aggression and feeding success of the anti-serum manipulated subdominant zebrafish, may result from a disturbed memory releasing it from its learned lower ranking status. Alternatively, the varied expression of ependymin protein across the different hierarchical levels may be linked to their different exposures to stress. Thus, the subdominants tend to be much more highly stressed because they participate continuously in aggressive interactions with the dominant that they inevitably lose. By contrast, the dominant fish tend to be less stressed, and the subordinate fish remove themselves from the competition by remaining passive and often sneakily obtaining food [4], [26]. The degree of stress experience might be in the order subdominant>dominant>subordinate which relates to the order of ependymin expression at the level of both transcript and protein. Stress has previously been linked to an increase in ependymin expression [32], [33], [35], and the gene expression profile presented here displays some evidence of stress effects, including up-regulation of hsp90 isoforms and down-regulation of DnaJ (hsp40) homologs. Future studies exploring manipulations of status linked to changes in ependymin should determine if ependymin expression are reversible when dominants lose their position or lower ranking individuals assert dominance.

The recognition that social status can be characterised at the level of the gene is very relevant given the manifold effects of social status on physiology. The divergent gene expression properties of individuals of different status levels undoubtedly contributes to the high variation observed between individuals held in batches of mixed animals whose social status is not defined. Accounting for social structure and dominance status within experimental designs is likely to offer significant improvements in the consistency of physiological characterisation and thus mechanistic interpretation. Knowledge of social position also allows responses to other treatments to be better characterised, and offers a better understanding of how environmental manipulations may affect the rank order. This is relevant to an understanding of the ecological and evolutionary importance of dominance hierarchies in nature and may assist us in understanding how hierarchies are established, how individuals achieve a specific status within a group and how this is maintained. Gene expression profiles may be to some extent controlled by the social relationships within a group thus providing some evidence for IGE models that predict an individual's genotype is also influenced by their interactions with conspecifics.

## Materials and Methods

### Animal Husbandry

All experiments were conducted in accordance with Home Office (UK) regulations and under local ethical approval. Juvenile male rainbow trout (n = 60; mean (±SE) weight  = 50.3 g ±1.0 g; length 159 mm (±2 mm)) were obtained from a commercial fish supplier and held for at least 2 weeks in stock tanks (2×2×0.5 m) with a recirculating filtration system supplied by aerated freshwater at 10±1°C, and fed daily with commercial trout pellets (Skretting, Northwich, UK) to 1.5% body weight per day. The fish were subjected to a 12:12 light:dark regime and provided with an opaque cover over half of the tank for shelter. Individuals of the same age and sex were used to avoid these confounding variables and juvenile rainbow trout readily form linear dominance hierarchies [6] which persists into adulthood.

### Stable hierarchies

Fish were individually removed from the stock tank at random and anaesthetised in benzocaine (0.05 g/L water), standard length measured using vernier callipers to 0.01 cm and weighed to 0.01 g. Each fish was tagged subcutaneously above the eye on either side using visible implant elastomer (VIE) tags to allow individual identification. These procedures do not compromise the growth, survival or behaviour of tagged individuals [36]. Six groups of three size-matched (±2%) rainbow trout were transferred to glass tanks (0.90×0.45×0.55 m) under the same conditions indicated above. The sides and rear of each tank were covered in opaque polythene and an opaque screen was placed over the front of the tank to minimise visual disturbance. After a settling period of one day, observations were made through a small opening in the opaque front screen using a low light level camera and video recording equipment. The behaviour of each group was recorded for 15 mins twice a day and food was introduced at a rate of 1.5% body weight per day during the observations (see File S1 for dominance measurement). All of the six groups displayed a clearly defined and consistent hierarchy over 7 days of observations. After 7 days, the fish were killed and the brain removed and stored at −80°C.

### Transitions in rank position

Hierarchies were set up as described, but after the 7 days of behavioural observations, the dominant was removed and replaced by a fish that was 30% smaller. This maintained constant the number of fish in each tank, and ensured that smaller individual just introduced would become the lowest ranked fish and that the rank 2, subdominant fish became rank 1, the dominant. Fish from each of six hierarchies were killed at either 2 hours, 48 hours and 1 week, and the brains were removed for transcript analysis (N = 6 per time point). During these time periods behaviour was assessed for 15 mins prior to sampling in the 2 hour treatment, and then daily for the 48 hour and 1 week time points, to confirm that stable linear hierarchies formed and the expected transition in status had indeed occurred.

### Microarray fabrication and analysis

Normalised cDNA libraries from trout brains, liver and skeletal muscle of control, hypoxia- cold- and warm-conditioned trout were prepared, cloned and EST characterized [37]. Amplicons were printed on Corning GAP2 slides using a BioRobotics TAS robot. For Experiment 1, the total RNA was separately isolated from the whole brain of each of the three ranked fish from each of 6 stable hierarchies, giving 18 RNA preparations. Brain RNA was also isolated from the new dominant specimen in each of the 6 manipulated hierarchies, for the 3 time points, giving a further 18 RNA preparations. A single reference mRNA was generated by pooling equimolar amounts of total RNA from all brain RNA preparations. RNA samples were labeled with Cy5 dyes (GE Healthcare, Amersham, UK) and hybridised under lifterslips (Implen, Munich, Germany) in hybridisation boxes (Genetix, New Milton, UK) against Cy3-labeled reference target. Dye-reversed arrays were also hybridized giving 72 arrays in total (see File S1 for labeling, hybridization, normalisation and statistical protocols). The raw array data has been deposited in a MIAME compliant database, the ArrayExpress repository (accession E-MAXD-33), and all data are MIAME compliant. The measured data was normalised through variance stabilised normalisation [38] followed by a LOWESS-based spot intensity dependent dye-bias correction [39] and a spatial bias correction. The normalised data was then entered into a linear model involving canonical parameters [40]. The Maximum Likelihood Estimation (MLE) of the parameters was generated by fitting the model to data. The significance of the estimated log-ratio values being different from the null value zero was determined using the t-test. The multiple testing problem was handled by using q-values [41] and differentially expressed (DE) genes were extracted by controlling the False Discovery Rate (FDR) [42], [43] at 10%, i.e. q<0.1. A second criterion was that fold change between two conditions exceeded 1.5. The resulting DE genes were clustered across all contrasts by the K-means method and the gene expression properties of all DE genes were visualised using heat-maps. Genes were BLASTx identified against a panel of databases as described previously [37] and for redundant clones a consensus expression profile was generated.

### RT-PCR confirmation of microarray data

We tested the validity of the expression values for genes obtained from the microarray experiment by undertaking real time-PCR (RT-PCR) of selected genes using the brain RNA samples used for the microarray experiment. The genes used were ependymin, GABA-receptor, Complement C3-1 and an unidentified gene (Gene X) with homologies to brain ESTs from other fish species. Expression of two housekeeping genes, GAPDH and 18S ribosomal RNA showed no significant difference between the rank members in the microarray analysis, so the 18S ribosomal RNA was used as the internal control (see Table S2 for primer information). All samples were further analysed by northern blot to confirm the RT-PCR data. The expression of ependymin, Gene X and GABA-RAP was analysed in the brains of individuals that made the transition from subdominant to dominant at all three time points to validate the array data (See File S1 for protocol and Figure S1).

### Analysis of protein expression

For the gene that showed the highest fold-change between the social ranks, namely ependymin, a western immunoblot was conducted. Three separate hierarchies were set up as described previously and verified for stability for 7 days after which the fish were killed and the brains were removed and stored at -80°C. Brain protein extracts were prepared following the methods of Pradel *et al*. [20] (see File S1 for protocol). The western blot procedure was also run using the pre-immune serum for a comparison with the immune serum and all samples were analysed using a rabbit anti-β tubulin polyclonal antibody as a control since the corresponding gene was not significantly different between the ranks in the microarray analysis described above.

### Zebrafish behavioural observations

Adult, male zebrafish, *Danio rerio*, (n = 60; mean weight 0.36±0.1 g) were obtained from a commercial supplier and held in two stock tanks (50×35×45 cm) each containing a gravel substratum, internal filter, aeration and held on a 12:12 h light:dark regime at 26±1°C. Adults were used as it is not easy to differentiate the sex of the individuals at juvenile stages, however, zebrafish adults are known to form easily quantifiable dominance relationships as adults [44] as well as at juvenile stages. Fish were fed daily using commercial fish flakes (Tetramin, Melle, Germany). Male fish were selected from one stock tank and size-matched to within 0.05 g with another male from the second stock tank and placed in pairs in glass observation tanks (30×15×20 cm) for 1 week to allow a stable dominant-subdominant relationship to form. Observations took place twice daily for 15 minutes, a.m. and p.m., for 3 successive days (see File S1 for behaviours recorded).

On day 4, each pair was assigned to one of the following treatment groups: Control (anaesthetised but no injection; n = 6), Buffer (phosphate-buffered saline (PBS), pH 7.4, containing 10 mM sodium phosphate, 120 mM NaCl, and 2.5 mM KCl injected intracerebroventricularly into both fish; n = 8); Subdominant (subdominant receives ependymin antisera (3 mg in 1 mL PBS), dominant injected with buffer only; n = 8); Dominant (dominant receives ependymin antisera (3 mg in 1 mL PBS), subdominant injected with buffer only; n = 6). We followed the well established intracerebroventricular injection method in the Schmidt laboratory in Giessen [19], [20]. Antisera was injected into the tectal brain ventricle by means of a Hamilton syringe (diameter 0.1 mm). Behavioural observations occurred 6 hours after the treatment and for the following 2 days as described above. All fish were humanely killed at the end of this experimental period.

## Supporting Information

Figure S1 **Changes in gene expression profile by manipulation of dominance status from subdominant to dominant.** Removal of the dominant specimen from stable hierarchy led to the adoption of dominant status by the previously sub-dominant individuals (N) within a hierarchy. Figure A illustrates the changes in brain transcripts of the new dominant (N) at 2h, 48h and 1 week (w), each being referenced against the dominant specimens (D) from the stable hierarchy. Each coloured line represents a different gene from the list of 110 differentially expressed genes. Figure B illustrates the same values for N referenced against the sub-dominant specimens from the stable hierarchy. Thus A illustrates the progressive adoption of the dominant profile over time, whilst B displays the divergence from the original sub-dominant profile.
(TIF)

Figure S2 **Western blotting of ependymin in stable rainbow trout dominance hierarchies.** An example of the protein expression using western blots to demonstrate ependymin is upregulated in the subdominant (Subdom.) and less is expressed by the dominant (Dom.) followed by the subordinate (Subord.). A housekeeping protein β tubulin is unchanged in expression for the 3 ranks.
(TIF)

File S1 **Details of experimental protocols including the behavioural observations, intracerebroventricular injections, microarray analysis; RT-PCR and western blot.**
(DOC)

Table S1 List of genes that were differentially expressed between sub-dominant and either dominant or subordinate members of a stable social hierarchy.
(DOCX)

Table S2 Primers for RT-PCR analysis.
(DOCX)
