# Supplementary figures and images for: Molecular Correlates of Social Dominance: A Novel Role for Ependymin in Aggression
Source: PLoS One. 2011 Apr 5;6(4):e18181. doi: 10.1371/journal.pone.0018181 (PMC3071721; doi:10.1371/journal.pone.0018181)

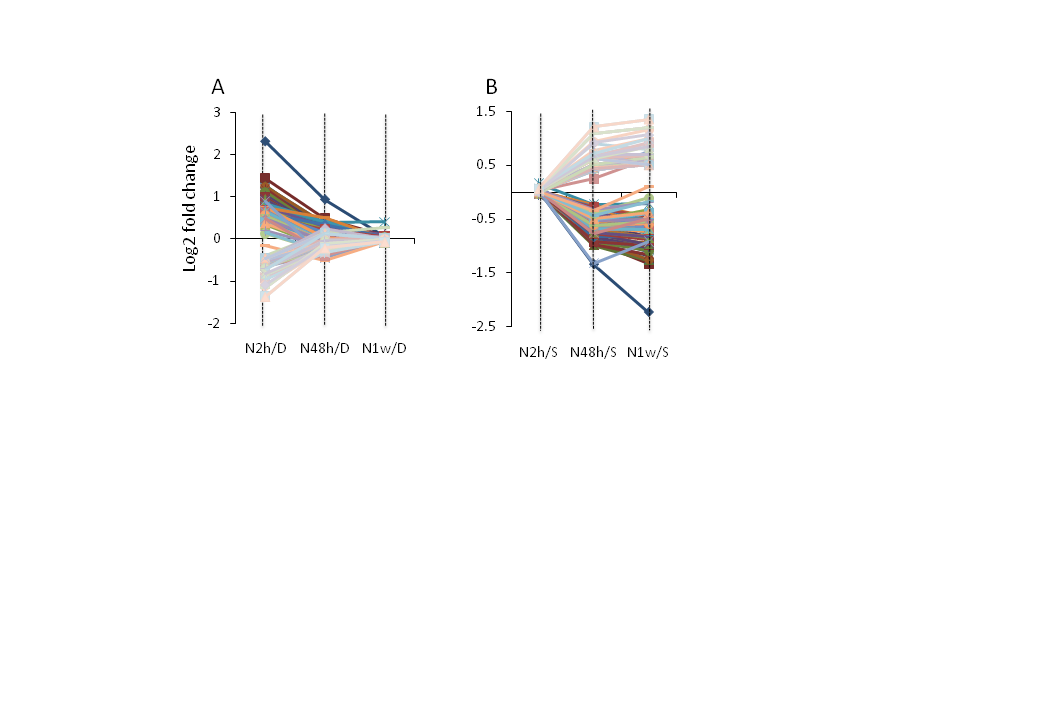

Supplement: Figure S1 — Changes in gene expression profile by manipulation of dominance status from subdominant to dominant. Removal of the dominant specimen from stable hierarchy led to the adoption of dominant status by the previously sub-dominant individuals (N) within a hierarchy. Figure A illustrates the changes in brain transcripts of the new dominant (N) at 2h, 48h and 1 week (w), each being referenced against the dominant specimens (D) from the stable hierarchy. Each coloured line represents a different gene from the list of 110 differentially expressed genes. Figure B illustrates the same values for N referenced against the sub-dominant specimens from the stable hierarchy. Thus A illustrates the progressive adoption of the dominant profile over time, whilst B displays the divergence from the original sub-dominant profile. (TIF) [file pone.0018181.s001.tif]

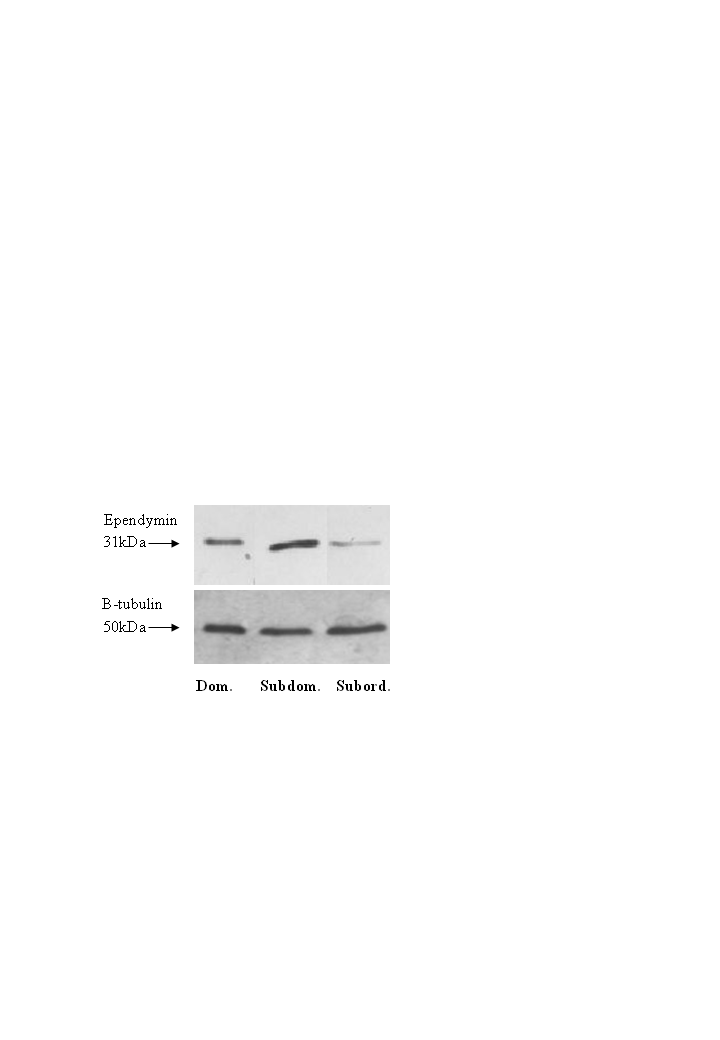

Supplement: Figure S2 — Western blotting of ependymin in stable rainbow trout dominance hierarchies. An example of the protein expression using western blots to demonstrate ependymin is upregulated in the subdominant (Subdom.) and less is expressed by the dominant (Dom.) followed by the subordinate (Subord.). A housekeeping protein β tubulin is unchanged in expression for the 3 ranks. (TIF) [file pone.0018181.s002.tif]
