# Supplementary material for: Molecular Correlates of Social Dominance: A Novel Role for Ependymin in Aggression
Source: PLoS One. 2011 Apr 5;6(4):e18181. doi: 10.1371/journal.pone.0018181 (PMC3071721; doi:10.1371/journal.pone.0018181)
Supplement: File S1 — Details of experimental protocols including the behavioural observations, intracerebroventricular injections, microarray analysis; RT-PCR and western blot. (DOC) [file pone.0018181.s003.doc]

**File S1: Supporting Information**

**MATERIALS AND METHODS**

***Behavioural Observations***

Rainbow trout: Behaviours noted for each individual were number of attacks, number of retreats and number of pellets eaten. A daily dominance score for each fish was calculated (frequency of attacks – frequency of retreats) allowing a linear hierarchy to be determined for each group for each experimental day; the fish with the highest score was accorded rank 1, the dominant, the fish with an intermediate score was rank 2, the subdominant, and finally the fish with the lowest score was rank 3, the subordinate.

Zebrafish: The following behaviours were recorded (i) frequency of attacks which was a direct and rapid movement of one fish towards the other fish and which resulted in a retreat or a counterattack; (ii) frequency of retreats which was a direct movement away from the attacking fish; and (iii) percentage of food obtained by each fish on adding 10 flakes to the tank.

***Intracerebroventricular injections***

Both fish were placed separately into large glass beakers (1 litre) containing water and anaesthetised by rapid chilling until loss of equilibrium and no reflex response to tail pinch. The buffer (10ul of 10mM NaPO4; 120mM NaCl; 2.5mM KCl, pH 7.4) was combined with the ependymin antisera to provide 200 pmol of antisera for the injections or buffer was used along at the same concentration. The relevant solution was injected into the tectal brain ventricle by means of a Hamilton syringe (diameter 0.1 mm) The injection site was the tectal ventricle, between the tectum opticum and the corpus cerebelli (see 9 for a detailed diagram). Each pair of fish were returned to their tank and were feeding normally within 15 minutes of recovery from anaesthesia.

***Microarray analysis***

Brain total RNA was isolated and purified by using Trizol (Sigma-Aldrich, UK, manufacturers instructions), quantified using a Nanodrop ND-1000 spectrophotometer (Wilmington, DE) and assessed for quality by electrophoresis. cDNA prepared from brain RNA was subjected to suppression subtraction hybridization, and cloned into pGEM(R)-T Easy vector (Promega) as described by Sneddon *et al.* (1). PCR amplicons of the resulting 5000 clones plus housekeeping genes were printed onto Corning GAP2 slides using a BioRobotics MicroGrid2 TAS robotic printer. The corresponding ESTs were assembled and annotated using and in-house informatics pipeline (*EST-Ferret*, see <http:legr.liv.ac.uk>) and the resulting searchable database (troutBASE2.0) can be found at <http:legr.liv.ac.uk>.

Fluorescence-labeled cDNA was produced by reverse transcription of brain total RNA in the presence of amino-allyl adducts followed by post-labeling with Cy3 and Cy5 dyes for the reference and individuals, respectively. Microarrays were hybridised with these labelled cDNA probes at 65°C overnight in sealed hybridisation chamber (Genetix, New Milton, UK). Fluorescent images from the 72 hybridised microarrays were captured using a Genepix 4000A laser scanning microscope and quantified using Genepix 3.0 software (both Molecular Dynamics, Sunnyvale, CA). The fluorescence data were expressed as normalized values of Cy5 and Cy3 fluorescence from each spot on the slides using GeneSpring software (Agilent, Santa Clara CA).

***RT-PCR***

RNA samples were treated with DNAse I (Invitrogen, Carlsbad, CA) following the manufacturers instructions and quantified using the Nanodrop spectrophotometer. Reverse transcription was performed using Superscript (Invitrogen) using a primer solution containing random hexamers and poly dT. The resulting cDNA was used in a quantitative real time PCR assay using a standard SYBR-GREEN RT-PCR protocol (Qiagen, Crawley, UK) in a Rotor-Gene RG 3000 real-time PCR machine (Corbett Life Sciences, Sydney, Australia). Each sample was tested in triplicate for each gene. Primers were designed using the online SIGMA primer design facility (<http://sigma-genosys.com/calc/DNAcalc.asp>) using the EST files from cDNA clones listed on troutBASE 2.0 at [http://legr.liv.ac.uk](http://legr.liv.ac.uk/)). Primer sequences are shown in Table S2.

***Western Blot***

Each brain was homogenised in 2 mL 50 mM Tris-buffered saline (TBS), pH 7.4, containing 90 mM NaCl, 2.5 mM CaCl_2_ with 1 mM glutathione and 0.3 mM N-tosyl-2-phenylalanine chloromethyl ketone as protease inhibitors for 30 min at 4°C. The samples were centrifuged at 13,000 rpm for 45 min (4°C) to obtain the soluble protein constituents of the supernatant. The homogenate was centrifuged at 1000 rpm (for 10 min at 4°C), and subsequently, the supernatant was centrifuged at 13,000 rpm (for 60 min at 4°C). The resulting pellet was resuspended in 0.3 mL TBS, and the supernatant was further centrifuged at 130,000 rpm (for 105 min at 4°C) to yield a supernatant containing the soluble cytoplasmic proteins.

The samples were separated by reducing polyacrylamide gel electrophoresis (SDS-PAGE, 10% crosslinked) (6). Prestained proteins (RPN800 Rainbow markers; GE Healthcare, Little Chalfont, UK) served as molecular weight standards. Proteins were transferred to nitrocellulose filters (7, 8) and immunostained with a polyclonal rabbit antibody directed against the ependymin peptide (H2N – QRI RVL EQK TGH TPC – CONH2) designed from the rainbow trout ependymin peptide sequence (UniProt Gene:EPD1_ONCMY; accession P28770) and manufactured by Eurogentec, Seraing, Belgium. Anti-ependymin was diluted in 10 mM Tris buffer, pH 7.4, containing 150 mM NaCl and 3% skimmed milk powder (TBSM) to a final concentration of 3 mg/mL. After preincubation in TBSM (for 12 h at 4°C) and incubation with anti-ependymin for 2 h at room temperature, nitrocellulose membranes were washed in TBSM containing 0.05% Nonidet P40 (Sigma, Poole, UK) and then incubated for 90 min with peroxidase-conjugated goat anti-rabbit- IgG (Sigma) diluted in TBSM. Nitrocellulose filters were then washed in TBS containing 0.1% Tween 20 (Sigma) and peroxidase activity was visualized by the enhanced chemiluminescence (ECL) protein detection system (GE Healthcare, Little Chalfont, UK).

**References**

1. Sneddon LU, Margareto J, Cossins AR (2005) The use of transcriptomics to address questions in behaviour: Production of a suppression subtractive hybridisation library from dominance hierarchies of rainbow trout. Physiol Biochem Zool 78: 695-705.
2. Benjamini Y, Hochberg D (2000) The adaptive control of the false discovery rate in multiple comparison problems. Ann Statist 29: 1165-1188.
3. Sloman KA, Metcalfe NB, Taylor AC, Gilmour KM (2001) Plasma cortisol concentrations before and after social stress in rainbow trout and brown trout. Physiol Biochem Zool 74: 383-389.
4. Gilmour KM, DiBattista JD, Thomas JB (2005) Physiological causes and consequences of social status in salmonid fish. Integr Comp Biol 45: 263-273.
5. Ward AJW, Webster MM, Hart PJB (2006) Intraspecific food competition in fishes. Fish Fisher 7: 231-261.
6. Laemmli UK (1970) Cleavage of structural proteins during assembly of head of bacteriophage-T4. Nature 227: 680-685.
7. Gershoni JM, Palade GE (1983) Protein blotting - principles and applications. Analyt Biochem 131: 1-15.
8. Towbin H, Staehelin T, Gordon J (1979) Electrophoretic transfer of proteins from polyacrylamide gels to nitrocellulose sheets - procedure and some applications. Proc Nat Acad Sci U S A 76: 4350-4354.
9. Pradel G, Schachner M, Schmidt R (1999) Inhibition of memory consolidation by antibodies against cell adhesion molecules after active avoidance conditioning in zebrafish. J Neurobiol 39: 197-206.
