# Supplementary material for: Molecular Correlates of Social Dominance: A Novel Role for Ependymin in Aggression
Source: PLoS One. 2011 Apr 5;6(4):e18181. doi: 10.1371/journal.pone.0018181 (PMC3071721; doi:10.1371/journal.pone.0018181)
Supplement: Table S1 — List of genes that were differentially expressed between sub-dominant and either dominant or subordinate members of a stable social hierarchy. (DOCX) [file pone.0018181.s004.docx]

**Table S1. List of genes that were differentially expressed between sub-dominant and either dominant or subordinate members of a stable social hierarchy.**

‘Clone i.d.’ indicates the plate/well number location for the cDNA gene probe (see legr.liv.ac.uk) with the corresponding GenBank accession and the accession and gene name for the top BLASTx hit. FDR indicates the calculated false discovery rate, and Cluster indicates which of the two clusters indicated in Figure 1 the gene probes belongs to. In ‘Gene Name’ the prefix ‘Predicted’ has been abbreviated to ‘P:’.

| Microarray probe identity | | BLASTx identity | | FDR | Cluster |
| --- | --- | --- | --- | --- | --- |
| Clone i.d. | Accession | Accession | Gene name |  |  |
|  |  |  |  |  |  |
| **Blood proteins, angiogenesis and immunity** | | |  |  |  |
| 06k07 | CT567780.1 | AAI63088.1 | Angptl3 protein | 0.01844 | 1 |
| 16c08 | CT565027.1 | NP_001117138.1 | β-globin | 0.01882 | 2 |
| 16e03 | CT564562.1 | NP_001118017.1 | β-globin | 0.00008 | 2 |
| 01e15 | CT567437.1 | NP_571877.1 | Ceruloplasmin | 0.00000 | 1 |
| 02p12 | CT567672.1 | AAG40610.1 | Complement C-3 | 0.04160 | 1 |
| 28n23 | CT569591.1 | AAB05029.1 | Complement component C3 | 0.01380 | 2 |
| 02b10 | CT568594.1 | NP_000022.3 | Delta-aminolevulinic acid dehydratase | 0.00658 | 1 |
| 06d20 | CT567097.1 | NP_997939.1 | Fibrinogen, B beta polypeptide | 0.01645 | 2 |
| 24p23 | HO666751 | AAG30004.1 | Haptoglobin fragment 1 | <0.00001 | 2 |
| 16c21 | CT564780.1 | XP_692783.2 | Similar to islet cell autoantigen 512 | <0.00001 | 1 |
| 10a03 | CT564568.1 | P02142.1 | Hemoglobin subunit beta-1 | 0.00001 | 1 |
| 22n10 | CT565441.1 | ABF57673.1 | Serum albumin | <0.00001 | 2 |
|  |  |  |  |  |  |
| **Cellular regulation** | |  |  |  |  |
| 16p11 | CT563640.1 | NP_001117944.1 | 14-3-3E1 protein | <0.00001 | 1 |
| 27c16 | CT568931.1 | NP_001134467.1 | Adenylate kinase | 0.00157 | 1 |
| 14h12 | CT564440.1 | NP_955864.1 | Calmodulin | <0.00001 | 1 |
| 16g16 | CT564115.1 | NP_998516.1 | Calmodulin 1a | 0.00001 | 1 |
| 16l15 | CT564197.1 | NP_999901.1 | Calmodulin 2, beta (phosphorylase kinase, delta) | <0.00001 | 1 |
| 16o17 | CT564455.1 | NP_892012.1 | Calmodulin 2, gamma | 0.00001 | 1 |
| 10p17 | CT564221.1 | NP_878278.1 | Cellular retinoic acid binding protein 1a | <0.00001 | 2 |
| 01o13 | CT566565.1 | ACH70843.1 | Glycogen synthase kinase binding protein-like | 0.00222 | 1 |
| 27i06 | CT569836.1 | ACH70652.1 | Phosphoglycerate kinase 1 | <0.00001 | 1 |
| 16k20 | CT565252.1 | NP_001026846.1 | Poly A binding protein, cytoplasmic 1 a | 0.00067 | 1 |
| 16d19 | CT563870.1 | XP_001919221.1 | P:Similar to calmodulin 2 | 0.00002 | 1 |
| 26p20 | CT569868.1 | XP_001922587.1 | P:Similar to copine V | 0.05637 | 2 |
| 24l12 | CT564474.1 | XP_700005.3 | P:Similar to Protein Shroom2(Protein APXL) | 0.00001 | 1 |
| 08o20 | CT566515.1 | NP_957283.1 | Protein kinase C and casein kinase substrate in neurons 3 | <0.00001 | 1 |
| 24l11 | CT565266.1 | NP_998458.1 | Protein phosphatase 2A, catalytic subunit, beta isoform | 0.03994 | 2 |
| 17f03 | CT568542.1 | NP_001133989.1 | Proteinase-activated receptor 2 | 0.00878 | 2 |
|  |  |  |  |  |  |
| **Cytoplasmic/cytoskeletal proteins** | | |  |  |  |
| 26l02 | CT570303.1 | NP_001001889.1 | Catenin, beta 2 | 0.00473 | 1 |
| 01k19 | CT567333.1 | ACI32952.1 | Claudin-3 | 0.00003 | 1 |
| 16n21 | CT563486.1 | NP_001118165.1 | Ependymin | <0.00001 | 1 |
| 16p12 | CT564351.1 | NP_001134381.1 | Ependymin-1 | <0.00001 | 1 |
| 16h16 | CT563302.1 | NP_957309.1 | ERGIC and golgi 3 isoform 2 | <0.00001 | 1 |
| 27l10 | CT569442.1 | NP_001117128.1 | Fast myotomal muscle tropomyosin | 0.00175 | 1 |
| 14k16 | CT565327.1 | ACI33945.1 | Myelin proteolipid protein | 0.00142 | 1 |
| 27c19 | CT570626.1 | NP_001117763.1 | Myosin light chain 1 | 0.00027 | 2 |
| 16a08 | CT563762.1 | XP_001919165.1 | P:Similar to cadherin 8, type 2 | 0.00554 | 1 |
| 26f09 | HO666752 | XP_001339206.2 | P:Similar to myosin heavy chain fast skeletal type 2 | 0.00001 | 1 |
| 14c03 | CT564826.1 | ACI67647.1 | Stathmin | 0.00003 | 1 |
| 22l02 | CT564140.1 | ACI32906.1 | Tubulin alpha chain | 0.03457 | 1 |
| 16m11 | CT564319.1 | NP_006073.2 | Tubulin, alpha, ubiquitous | 0.01919 | 1 |
|  |  |  |  |  |  |
| **Energy pathways** | |  |  |  |  |
| 14l12 | CT565248.1 | ACH85277.1 | ATP synthase H+ transporting mitochondrial F1 complex β | 0.00011 | 1 |
| 14j11 | CT564124.1 | ACM80354.1 | ATP synthase H+ transporting mitochondrial F1 complex γ | 0.00055 | 1 |
| 21k22 | HO666746 | NP_775329.1 | Creatine kinase b, brain | <0.00001 | 1 |
| 11a15 | HO666744 | NP_059334.1 | Cytochrome c oxidase subunit II | 0.02615 | 2 |
| 01m07 | CT566639.1 | NP_998297.1 | Fructose-1,6-bisphosphatase 1b | 0.01150 | 1 |
| 27f05 | CT569598.1 | ABN54436.1 | Glyceraldehyde-3-phosphate dehydrogenase | 0.00097 | 1 |
| 27k04 | CT568898.1 | ACH70936.1 | Glyceraldehyde-3-phosphate dehydrogenase-1 | 0.00008 | 1 |
| 14p11 | CT564516.1 | NP_956335.1 | Mitochondrial ATP synthase gamma-subunit | 0.00001 | 1 |
| 27n20 | HO666753 | NP_957318.1 | Phosphoglycerate mutase 2 | <0.00001 | 1 |
| 27e15 | CT570381.1 | NP_001133202.1 | Phosphoglycerate mutase 2-2 (muscle) | 0.00001 | 1 |
| 16a17 | CT565676.1 | NP_001076267.1 | Tyrosine 3/tryptophan 5-monooxygenase activation protein, β | <0.00001 | 1 |
| 14f19 | CT564330.1 | NP_006752.1 | Tyrosine 3/tryptophan 5-monooxygenase activation protein, ε | 0.01962 | 1 |
|  |  |  |  |  |  |
| **Intermediary and lipid metabolism** | | |  |  |  |
| 14f14 | CT563584.1 | NP_001134589.1 | Acyl-CoA-binding domain-containing protein 7 | 0.00005 | 2 |
| 14i11 | CT564274.1 | NP_001134834.1 | Apolipoprotein C-I | <0.00001 | 1 |
| 24i08 | HO666748 | AAG30007.1 | Biotinidase fragment 1 | <0.00001 | 2 |
| 19p24 | CT566429.1 | AAM73701.1 | C1q-like adipose specific protein | <0.00001 | 1 |
| 16l22 | HO666745 | NP_571924.2 | Alcohol dehydrogenase, Class III, chi subunit | 0.00036 | 1 |
| 06g21 | CT566808.1 | NP_956537.1 | Glycerophosphodiester phosphodiesterase 1 | 0.00002 | 2 |
| 10n08 | CT563751.1 | XP_001144055.1 | P: UDP glycosyltransferase 2 family, polypeptide B11 | 0.00032 | 1 |
| 27m12 | CT568892.1 | NP_001046.2 | sulfotransferase family, cytosolic, 1A, phenol-preferring | 0.02304 | 1 |
| 26o22 | CT569501.1 | NP_066962.2 | UDP glucuronosyltransferase 2B4 precursor | <0.00001 | 1 |
|  |  |  |  |  |  |
| **Regulation of nuclear processes** | | |  |  |  |
| 14b18 | CT563836.1 | ACM09235.1 | High mobility group-T protein | 0.00004 | 1 |
| 27c06 | CT570403.1 | NP_001133170.1 | High-mobility group box 2 | 0.00003 | 1 |
| 01g08 | CT567400.1 | NP_004417.2 | Polyhomeotic 1-like | 0.03599 | 1 |
| 14o09 | CT565057.1 | NP_004080.2 | TSC22 domain family, member 3 isoform 2 | 0.00025 | 2 |
| 09c13 | HO666741 | NP_060853.3 | WD repeat domain 33 isoform 1 | <0.00001 | 2 |
|  |  |  |  |  |  |
| **Protein turnover** | |  |  |  |  |
| 08g19 | CT568195.1 | ACN60319.1 | Cathepsin Z precursor | 0.00001 | 2 |
| 14h21 | CT565586.1 | ACI34318.1 | Cytosolic non-specific dipeptidase | 0.00567 | 1 |
| 26n02 | CT569014.1 | NP_001952.1 | Eukaryotic translation elongation factor 2 | 0.00030 | 1 |
| 17d04 | CT566446.1 | NP_001018424.1 | Inter-alpha (globulin) inhibitor H3 | 0.00021 | 1 |
| 16n06 | CT564987.1 | NP_956083.1 | Proteasome 26S subunit, non-ATPase, 7 | 0.01728 | 1 |
| 14p15 | CT564964.1 | NP_001029025.1 | Ribosomal protein L3 isoform b | <0.00001 | 1 |
| 09a08 | CT563257.1 | NP_001035132.1 | Ribosomal protein L32 | <0.00001 | 2 |
| 10h18 | CT564525.1 | NP_001002069.1 | Ribosomal protein L37 | <0.00001 | 1 |
| 25n02 | CT569731.1 | NP_956341.1 | Ribosomal protein L7a | 0.00016 | 1 |
| 14n12 | CT564656.1 | NP_150644.1 | Ribosomal protein L8 | 0.01175 | 1 |
| 16n05 | CT563291.1 | NP_001001819.1 | Ribosomal protein S15 | <0.00001 | 1 |
| 14i19 | CT563846.1 | NP_001012.1 | Ribosomal protein S17 | <0.00001 | 1 |
| 16l13 | CT565306.1 | NP_001019.1 | Ribosomal protein S25 | <0.00001 | 1 |
| 03c23 | HO666740 | NP_001108176.1 | Serpin peptidase inhibitor | 0.07375 | 1 |
| 06g14 | CT567269.1 | ACI68896.1 | Translocon-associated protein subunit delta precursor | 0.00008 | 2 |
|  |  |  |  |  |  |
| **Systems regulation** | |  |  |  |  |
| 16j19 | CT565172.1 | ACM09767.1 | Pleiotrophic factor-alpha-2 precursor | <0.00001 | 1 |
| 16m15 | CT565177.1 | ACM08274.1 | Pleiotrophin precursor | 0.00024 | 1 |
| 14p22 | CT564977.1 | XP_001332909.2 | P: similar to GABA receptor associated protein | <0.00001 | 1 |
| 16o19 | CT565003.1 | NP_956021.1 | Tumor differentially expressed 2 | 0.00011 | 1 |
|  |  |  |  |  |  |
| **Stress proteins** | |  |  |  |  |
| 24l09 | CT565769.1 | NP_955904.1 | DnaJ (Hsp40) homolog, subfamily C, member 3 | <0.00001 | 2 |
| 22m17 | CT564070.1 | NP_001134029.1 | DnaJ homolog subfamily C member 3 | 0.02329 | 2 |
| 16o18 | CT563875.1 | NP_001118063.1 | Heat shock 90kDa protein 1 beta isoform b | 0.00182 | 1 |
| 14e15 | CT564853.1 | NP_571385.2 | Heat shock protein 90kDa alpha, class B member 1 | 0.00263 | 1 |
| 14m13 | CT565690.1 | NP_001117004.1 | Heat shock protein hsp90 beta | 0.00021 | 1 |
|  |  |  |  |  |  |
| **Transporters** | |  |  |  |  |
| 25d21 | CT570911.1 | NP_004311.1 | ATPase, Ca++ transporting, fast twitch 1 isoform b | 0.01186 | 2 |
| 27h24 | CT569775.1 | NP_001672.1 | ATPase, Ca++ transporting, slow twitch 2 isoform 2 | 0.00001 | 2 |
| 14o24 | CT563306.1 | NP_001117931.1 | Na/K ATPase alpha subunit isoform 1c | <0.00001 | 1 |
| 16p09 | CT564248.1 | ACN11046.1 | Sodium/potassium-transporting ATPase subunit beta-233 | <0.00001 | 1 |
|  |  |  |  |  |  |
| **Miscellaneous** | |  |  |  |  |
| 03b18 | CT565861.1 | XP_001661219.1 | Hypothetical protein AaeL_AAEL010978 | 0.04326 | 2 |
| 14o08 | CT564573.1 | NP_001118186.1 | Hypothetical protein LOC100136765 | <0.00001 | 1 |
| 08j24 | CT566501.1 | NP_001003460.1 | Hypothetical protein LOC445066 | 0.04201 | 1 |
| 11a09 | HO666743 | XP_001407819.1 | Hypothetical protein MGG_12193 | 0.00268 | 2 |
| 14k12 | CT564095.1 | YP_317745.1 | Hypothetical protein Nwi_1131 | 0.00038 | 1 |
| 06k06 | CT566625.1 | NP_998087.1 | OTU domain containing 3 | 0.00001 | 2 |
| 25p10 | CT570340.1 | XP_689811.2 | P: hypothetical protein LOC553498 | 0.00143 | 1 |
| 27d06 | CT569633.1 | XP_001924014.1 | P: ns:zf-e68 | 0.00598 | 1 |
| 09m18 | HO666742 | NP_113687.2 | Proline rich protein 2 | <0.00001 | 2 |
| 24p02 | HO666750 | NP_005030.2 | Proline-rich protein BstNI subfamily 1 isoform 1 preproprotein | <0.00001 | 2 |
| 22p17 | HO666747 | NP_955386.1 | Proline-rich protein BstNI subfamily 1 isoform 3 preproprotein | 0.00003 | 2 |
| 24l21 | HO666749 | NP_006239.3 | Proline-rich protein BstNI subfamily 2 | <0.00001 | 2 |
| 02j15 | HO666739 | CAD58854.1 | Putative extensin precursor | 0.00082 | 2 |
| 08k01 | CT567300.1 | NP_001119862.1 | Similar to polytropic murine leukamia virus receptor SYG1 | <0.00001 | 1 |
| 17g01 | CT566086.1 | YP_001469853.1 | SIS domain-containing protein | 0.00003 | 1 |
|  |  |  |  |  |  |
|  |  |  |  |  |  |
