# Supplementary material for: Molecular Correlates of Social Dominance: A Novel Role for Ependymin in Aggression
Source: PLoS One. 2011 Apr 5;6(4):e18181. doi: 10.1371/journal.pone.0018181 (PMC3071721; doi:10.1371/journal.pone.0018181)
Supplement: Table S2 — Primers for RT-PCR analysis. (DOCX) [file pone.0018181.s005.docx]

**Table S2.** **Primers for RT-PCR analysis.**

Forward Reverse

Ependymin TGA GGG GAA CAA TCA GAC TCG TGA

TGA AAG TG GTG GCA TC

GABARAP GGG AAG TCC TCA TGG GAC GGG CTC

AGC TGC ATT TTG GAT

MAPK TGC CTG GAC AGT TCC TTC CGA GAA

GCA GAA TCC GCG TGA TAG

Complement C3-1 GGC CAG TCC CTG GGT GGA CTG TGT GGA

TGG TTA TCC GTA

GAPDH CTG ATC GTT GAT ATC ACA AAC ATG GGG

GGT CAG GC TGC GTC

18s ribosomal AAA CGG CTA CCA CAC CAG ATT TGC CCT

protein CAT CCA AG CCA
